# Community Assessment of COPD Health Care (COACH) study: a clinical audit on primary care performance variability in COPD care

**DOI:** 10.1186/s12874-018-0528-4

**Published:** 2018-07-03

**Authors:** María Abad-Arranz, Ana Moran-Rodríguez, Enrique Mascarós Balaguer, Carmen Quintana Velasco, Laura Abad Polo, Sara Núñez Palomo, Jaime Gonzálvez Rey, Ana María Fernández Vargas, Antonio Hidalgo Requena, Jose Manuel Helguera Quevedo, Marina García Pardo, Jose Luis Lopez-Campos, on behalf of the COACH study investigators, Jose Luis Lopez-Campos, Ana Morán Rodríguez, Enrique Mascarós Balaguer, María Abad Arranz, Ana M. Fernández Vargas, Antonio Hidalgo Requena, José Manuel Helguera Quevedo, Sara Núñez Palomo, Ana María Fernández Vargas, Beatriz Elorza Maza, Cristina Pulido Vázquez, Eloy de Haro Muñoz, Enrique Pérez Ortiz, Fernando López Verde, Francisco José Guirado Hidalgo, Irene Martínez Ríos, Jose María Lopez López, Juan Antonio Lara González, Juan Carlos Navarro González, Manolo Del Rey Pérez, Manuel Resa Alcalá, Ramon Nicolas Franco Reins, Raúl Rabaneda Bueno, José Galván Espinosa, José Miguel Cerón, Antonio Núñez Montenegro, Eugenio Contreras, Andra Crisán, Bárbara Quevedo Benyto, Beatriz Sanchís Yago, Carmen Quintana Velasco, Clara Cañardo Alastuey, Clara Lanuzzelli Barroso, Clara López Mas, Daniel Rubio Castro, Laura Abad Polo, Marina García Pardo, Arancha Rojo Calderón, Carmen Toca Incera, Esperanza Rueda Alonso, Helena Bermejo Ruiz, Juan Carlos López Caro, Luis Sainz de Rozas Arpide, María Jesús Fernández Lerones, Jaime Gonzálvez Rey, Gabriel Romero de Ávila Cabezón, Carlos Chapela Villa, Ruth Otero González, Jose Ramón Parada Jorgal, Irene Valle González, Maite Espantoso Romero, Susana Friande Pereira, Alejandra Montero Costa, Alicia Jorge Formariz, Ana Isabel Hernández Regalado, Ana Maria Rey López, Ángel Alonso Val, Ángel Luis Laguna, Ángel Quijada Monso, Ángeles Casado Aguado, Beata Bordzynska Michalska, Beatriz Orozco Carratalá, Beatriz Solans Aisa, Carmen Mateo Pascual, Carmen Vieira Pascual, Cristina Alvarez Alonso, Cristina García Lombardia, David González Gallardo, Dolores Morata Perelló, Eleonora Valinoti, Elisabet Astorga García, Encarnación Santos Montealegre, Enrique Rodríguez de Mingo, Francisco Sánchez Martín, Gloria Viñas Fernández, Guillermina López Fernández, Itziar Valero Infantes, Jaime Marín Cañada, José Luis Martínez Carrasco, José Ramón Sánchez Picón, Juan Carlos Moreno Fernández, Juan Miguel Pinar Manzanet, Julia Caballer Rodilla, Juncal Martínez Irazusta, Leticia Pérez Esteban, Luis Toledano Rubio, Maria Belén García Benito, Maria Belén Ortega Trompeta, María Cruz Díez Pérez, María Dolores Tuñón Leiva, María José Lougedo Calderón, María Sol Lorenzo Borda, Margarita Jimeno Rodríguez, Maria Carmen Lobón Agúndez, Maria Dolores García Granado, Marta Ruesga Estébanez, Mercedes Adriaá Sanz, Mercedes Capitán Caldas, Mercedes Marinas Barba, Miguel Ángel Delgado Nicolás, Miriam Sánchez Herraínz, Mirian Sánchez Herráiz, María Belén Martinez Urroz, María del Pino Calderín Morales, Montserrat Uriel Martínez, Norma Doria Carlín, Nuria de la Peña Antón, Paloma López-Hermosa Seseña, Pilar Aranda Arias, Pilar Blanco García, Raquel García Arriola, Raquel Martínez Bernardos, Raquel Sanjurjo Navarro, Raúl de Simón Gutiérrez, Sergio Heras Criado, Sonia Carmon aGranados, Teresa Gómez Rodríguez, Teresa Troyano Rivas

**Affiliations:** 1 Unidad Médico-Quirúrgica de Enfermedades Respiratorias, Instituto de Biomedicina de Sevilla (IBiS), Hospital Universitario Virgen del Rocío/Universidad de Sevilla, Avda. Manuel Siurot, s/n, 41013 Seville, Spain; 2 UGC-DCCU Bahía de Cádiz-La Janda, Cadiz, Spain; 3 Centro de Salud Fuente de San Luis, Valencia, Spain; 4 Centro de Salud Perpetuo Socorro, Huesca, Spain; 5 Centro de Salud Illueca, Sector Calatayud, Zaragoza, Spain; 6 Centro de Salud Torrelaguna, Madrid, Spain; 7 Centro de Salud Matamá, Vigo, Spain; 8 Centro de Salud La Victoria, Málaga, Spain; 9 Centro de Salud de Lucena, Córdoba, Spain; 10 Centro de Salud Bajo Asón, Ampuero, Cantabria Spain; 11 Centro de Salud de Inca, Mallorca, Spain; 12 Centro de Investigación Biomédica en Red de Enfermedades Respiratorias (CIBERES), Instituto de Salud Carlos III, Madrid, Spain

**Keywords:** COPD, Clinical audit, Primary care, Quality of care, Variability

## Abstract

**Background:**

A thorough evaluation of the adequacy of clinical practice in a designated health care setting and temporal context is key for clinical care improvement. This study aimed to perform a clinical audit of primary care to evaluate clinical care delivered to patients with COPD in routine clinical practice.

**Methods:**

The *Community Assessment of COPD Health Care* (COACH) study was an observational, multicenter, nationwide, non-interventional, retrospective, clinical audit of randomly selected primary care centers in Spain. Two different databases were built: the resources and organization database and the clinical database. From January 1, 2015 to December 31, 2016 consecutive clinical cases of COPD in each participating primary care center (PCC) were audited. For descriptive purposes, we collected data regarding the age at diagnosis of COPD and the age at audit, gender, the setting of the PCC (rural/urban), and comorbidities for each patient. Two guidelines widely and uniformly used in Spain were carefully reviewed to establish a benchmark of adequacy for the audited cases. Clinical performance was analyzed at the patient, center, and regional levels. The degree of adequacy was categorized as excellent (> 80%), good (60–80%), adequate (40–59%), inadequate (20–39%), and highly inadequate (< 20%).

**Results:**

During the study 4307 cases from 63 primary care centers in 6 regions of the country were audited. Most evaluated parameters were judged to fall in the inadequate performance category. A correct diagnosis based on previous exposure plus spirometric obstruction was made in an average of 17.6% of cases, ranging from 9.8 to 23.3% depending on the region. During the audited visit, only 67 (1.6%) patients had current post-bronchodilator obstructive spirometry; 184 (4.3%) patients had current post-bronchodilator obstructive spirometry during either the audited or initial diagnostic visit. Evaluation of dyspnea was performed in 11.1% of cases. Regarding treatment, 33.6% received no maintenance inhaled therapies (ranging from 31.3% in GOLD A to 7.0% in GOLD D). The two most frequently registered items were exacerbations in the previous year (81.4%) and influenza vaccination (87.7%).

**Conclusions:**

The results of this audit revealed a large variability in clinical performance across centers, which was not fully attributable to the severity of the disease.

## How this fits in

To the best of our knowledge, this is the first ever clinical audit of primary clinical care delivery to COPD patients in Spain. The results of this study indicate that there is considerable variability in clinical performance, not completely attributable to the severity of the disease. Identification of the determinants of this variability will help us understand clinical behavior, and establish strategies to strengthen clinical practice for COPD in primary care.

## Background

Measurement of the adequacy of clinical practice in a designated health care setting and temporal context is key for clinical care improvement. In this regard, clinical audits provide an innovative tool to assess the quality of care, playing a significant role in highlighting the gaps between the recommended practices and the care that patients actually receive [1]. Accordingly, audits and feedback have shown to improve health care for different disease conditions [2].

Chronic obstructive pulmonary disease (COPD) is a chronic respiratory disease with extrapulmonary implications that poses a major burden for the patient and the health system [3, 4], with a high prevalence [5], morbidity and mortality [6] rate. Hence, the care for these patients should engage the highest quality standards due to its potential impact on the lives of patients, and relatives, alongside the strain on resources. Therefore, COPD is one of the diseases in which clinical audits are deemed to be of special relevance.

Until the last few years, clinical audits for COPD were not frequently carried out. Over the last several decades, the United Kingdom [7], followed by Spain [8, 9], have been leading the audit process for COPD in Europe. Additionally, several countries have recently started their own audit projects [10–12], like the recent European Clinical COPD Audit in 13 European countries [13, 14]. In Spain, the so-called AUDIPOC network evaluated clinical care in patients hospitalized due to COPD [15] and recent initiatives have explored the clinical performances in specialized respiratory outpatient clinics [16]. These audits have provided valuable information about medical interventions in hospital wards for patients admitted with COPD exacerbation [14], the resources available [17], and the interrelationship between resources and clinical practice [15, 16]. Although not formally labelled as clinical audits, previous analyses have been done describing different aspects of clinical performance in primary care using different methodological approaches [18–20].

The information regarding how COPD patients are treated in the primary care setting is essential and would provide very relevant information on the process of care. It might also reveal the key areas wherein improvements are required in order to complete the picture obtained in secondary care audits. Therefore, formal clinical audits in primary care are emerging to evaluate clinical performance in this setting using a standardized methodology (https://www.rcplondon.ac.uk/COPD). Based on our previous audit experiences, we have developed a clinical audit for COPD patients in the primary care setting in Spain. In this manuscript, we describe the full methodology and main results of this clinical audit regarding guideline adherence in primary care centers (PCC) in Spain. The results of this audit will serve as a basis for an improvement in health care delivery for COPD patients.

## Methods

The *Community Assessment of COPD Health Care* (COACH) study was an observational, cross-sectional, multicenter, national, retrospective, and non-interventional clinical audit aimed at evaluating the clinical care delivered to COPD patients in randomly selected primary care centers in Spain. This is the result of a joint project between the Spanish Primary Care Respiratory Group (GRAP) and the Spanish Society of Pneumology and Thoracic Surgery (SEPAR). The enrolment of participants and data collection was performed retrospectively.

### Governance

The COACH study was managed by a steering committee comprising two primary care physicians and two pulmonologists, with expertise in COPD care and clinical audits. One of them acted as a project manager for the daily oversight of the project. Each participating region selected a primary care physician to act as regional project coordinator. Altogether, the steering committee including the project manager and all regional representatives formed the expert panel, an operational group that was responsible for ensuring the success of the data collection and which provided feedback on the process and suggested improvements through regular face-to-face meetings and teleconferences. Within each participating region, a number of PCC were selected, and primary care physicians were appointed as local investigators responsible for local data collection on patients and the organization of care.

### Selection of centers

A list of all PCC in Spain was obtained from the official web pages of regional health systems in the country and entered into an Excel spreadsheet (Microsoft Corporation, Redmond, WA, USA). Since the size of each region and their respective number of provinces varied, the arbitrary proportion of PCC to be selected in each province was 10%, as determined by the expert panel. The Excel spreadsheet was thus programmed to randomly select 10% of the PCC in every province. Each regional representative was responsible to contact their respective selected centers to explain the project, offer participation, and obtain the final local approval. If any center rejected participation, this was replaced by the next center on the randomization list.

### Data item selection

Two different databases were built: the resources and organization database for recording information on the availability of resources in the PCC regarding COPD care, and the clinical database for recording clinical information from audited cases. The steering committee created the first draft of the databases, which were further revised in a face-to-face kick-off meeting that took place in Madrid on September 13th, 2014, and by email and teleconferences thereafter to refine the final version.

For descriptive purposes, we collected data on age at diagnosis of COPD and at audit, gender, setting of the PCC (rural/urban), and comorbidities. Rural areas were defined as areas with a population of less than 25,000. Comorbidities were evaluated by Charlson [21] and a COPD specific comorbidity (COTE) [22] indexes. To further characterize the comorbidities, we classified them into four groups, namely, cardiac conditions (including coronary artery disease, heart failure and atrial fibrillation or flutter), vascular conditions (including coronary artery disease, peripheral vascular disease, and cerebrovascular disease), neoplasms (including any solid or non-solid neoplasms of any origin), and psychiatric medication use (anxiolytics, antidepressants, and antipsychotics). Moreover, some other comorbidities of clinical relevance were recorded including benign prostate hyperplasia as a risk factor for urinary retention requiring the use of antimuscarinic drugs [23], and use of eye drops as a marker of eye conditions [24].

For audit evaluation purposes, two guidelines widely and uniformly used in Spain – the Global Initiative for Obstructive Lung Disease (GOLD) guideline [25] and Spanish National Guideline for COPD (GesEPOC) [26] were carefully reviewed to establish a benchmark for the adequacy of care in the audited cases. Only items regarding diagnosis and treatment of stable COPD were considered. Therefore, exacerbation care was not evaluated in this audit. The items considered to reflect good practices from a diagnosis and evaluation perspective were: an accurate diagnosis, the evaluation of symptoms, the number of exacerbations and hospitalizations (since they are relevant in selecting the appropriate interventions according to current guidelines), and the treatment strategy. Symptoms evaluated were dyspnea measured by the Medical Research Council (MRC) scale [27], the presence of chronic productive cough, sputum color in stable disease and the evaluation of asthma symptoms (as defined by the Global Initiative for Asthma [GINA] [28] including wheeze, shortness of breath, chest tightness, cough that occurs variably over time and varies in intensity, is often worse at night or on walking, triggered by exercise, laughter, allergens, or cold air, and often appears or worsens with viral infections). A record of the current pharmacological and non-pharmacological therapeutic scheme, adverse events, compliance, and inhaler satisfaction were also judged to be of relevance during the clinical interview of COPD patients. Inhaler satisfaction did not specifically reflect the correct inhaler technique, but was extracted from the clinical records if the general satisfaction of the patient with the inhaler was recorded.

The accuracy of the diagnosis was confirmed by previous exposure to inhalational irritants plus a post-bronchodilator non-reversible obstruction on spirometry. Information regarding previous exposure to noxious particles or gases included a history of active smoking, passive smoking, occupational exposure, biomass exposure or any other form of exposure judged to be relevant to the diagnosis as reflected in the medical record. Limited reversal of airflow was assessed by post-bronchodilator spirometry; however, after the first evaluation we noted that most patients had only a pre-bronchodilator spirometry. Therefore, we used pre-bronchodilator values for those cases without post-bronchodilator spirometry values. The obstruction also had to be present during the last recorded visit to consider it non-reversible.

From a therapeutic point of view, the use of a long acting bronchodilator (LABD), either a long-acting ß_2_ agonist (LABA) or a long-acting muscarinic antagonist (LAMA), as the basis for pharmacological treatment, the compliance with the GOLD recommendations in pharmacological prescription, adequate recommendation on non-pharmacological treatments including advising to quit smoking, vaccine recommendations, and exercise recommendations, were recorded.

For the adequacy of maintenance inhaled therapies, we assumed the following scenarios to reflect incorrect prescriptions: GOLD 2017 B-D patients without any medication recorded, any patient on inhaled corticosteroids (ICS) alone, and those treatment plans that duplicated drugs in combined and single therapies.

### Protocol of the study

Information was recorded by an ad-hoc built database via a centralized web page. A freelance web programmer with experience in database management was commissioned to create this website and the structure of the database. The website was organized as a hierarchical tool with different levels of responsibilities and rights to process data. Only members of the steering committee had full access to all data and the right to process them. At the regional level, there was a hierarchical access for regional administrators down to the level of local investigators coordinating the local data collection. To minimize a potential bias due to several auditors evaluating different medical records from different systems, we trained the auditors on how to proceed during data gathering and established limitations and rules in the database.

Data was collected from January 1, 2015 to December 31, 2016. The doctors in charge of the patients were unaware of the audit. During this period, the local investigator consecutively reviewed the medical records for confirmed cases of COPD and recorded the audit information for 80 cases per PCC. Accordingly, the selection of cases was based on a registered diagnosis of COPD in the medical record. No diagnostic criteria were considered for inclusion, since assessing the quality of the diagnoses was an outcome of the audit. The classification of patients as having COPD within the medical records was sufficient to include them in the audit. No exclusion criteria were defined.

### Ethical considerations

The study complied with the ethical requirements of the Helsinki Declaration regarding studies involving human subjects and with the Spanish regulation on data protection and confidentiality (Spanish Organic Law 15/1999 of December 13, on the Protection of Personal Data). The study was evaluated by the Spanish Agency for Medicine and Health Products and classified as a not post-commercialization observational study. The main ethical approval was obtained from the regional ethical committee of Andalusia region (approval number 02/2014) and any other participating region if required based on regional legislation. The clinical records were anonymized in the database by assigning a numerical code through an algorithm. No personal information that could be used directly or indirectly to identify an individual was entered. The relationship between the audit code and the clinical history number was kept locally, being the responsibility of the local investigator to avoid duplications. Because of the retrospective nature of the study, the anonymization of data, and the lack of active research interventions, the need for informed consent was waived. The ethics committee was aware of these circumstances clearly explained in the protocol, and approved this procedure.

### Statistical analysis

All computations were performed using SPSS Statistics version 20.0 (IBM Corporation, Armonk, NY, USA). Before performing any analysis, the database was evaluated for quality. The values that were extreme from a clinical point of view, missing, or inconsistent were returned to the investigators for correction. The clinical variables are presented as means and standard deviations or absolute and relative frequencies, as appropriate, at the patient level. The variability was expressed by using the inter-regional range, which represents the highest and lowest mean values from the participating center at the regional level, and the inter-center range, representing the highest and lowest mean values from the audited cases at the PCC level. Five centers with less than 10 included cases were not considered in calculating the inter-center range. Due to the low number of cases in the Balearic Islands, they were not considered in the calculation of the inter-regional range. The significance of this variability among the different participating centers and regions was explored for quantitative variables by the ANOVA test, after checking the equality of the variances with the Levene test. If the homoscedasticity assumption was not met, we used the Welch test. For the analysis of the categorical variables, we used the Chi-square test. The alpha error was set at 0.05 (two-tailed).

Based on previous experiences by our group [16], the degree of adequacy between the audited information and the recommendations of the consulted documents was categorized as excellent (> 80%), good (60–80%), adequate (40–59%), inadequate (20–39%), and highly inadequate (< 20%). A subgroup analysis was performed on those cases providing data on disease subtypes according to GOLD 2017 patient types to explore the distribution of treatments among them.

## Results

**Fig. 1:**
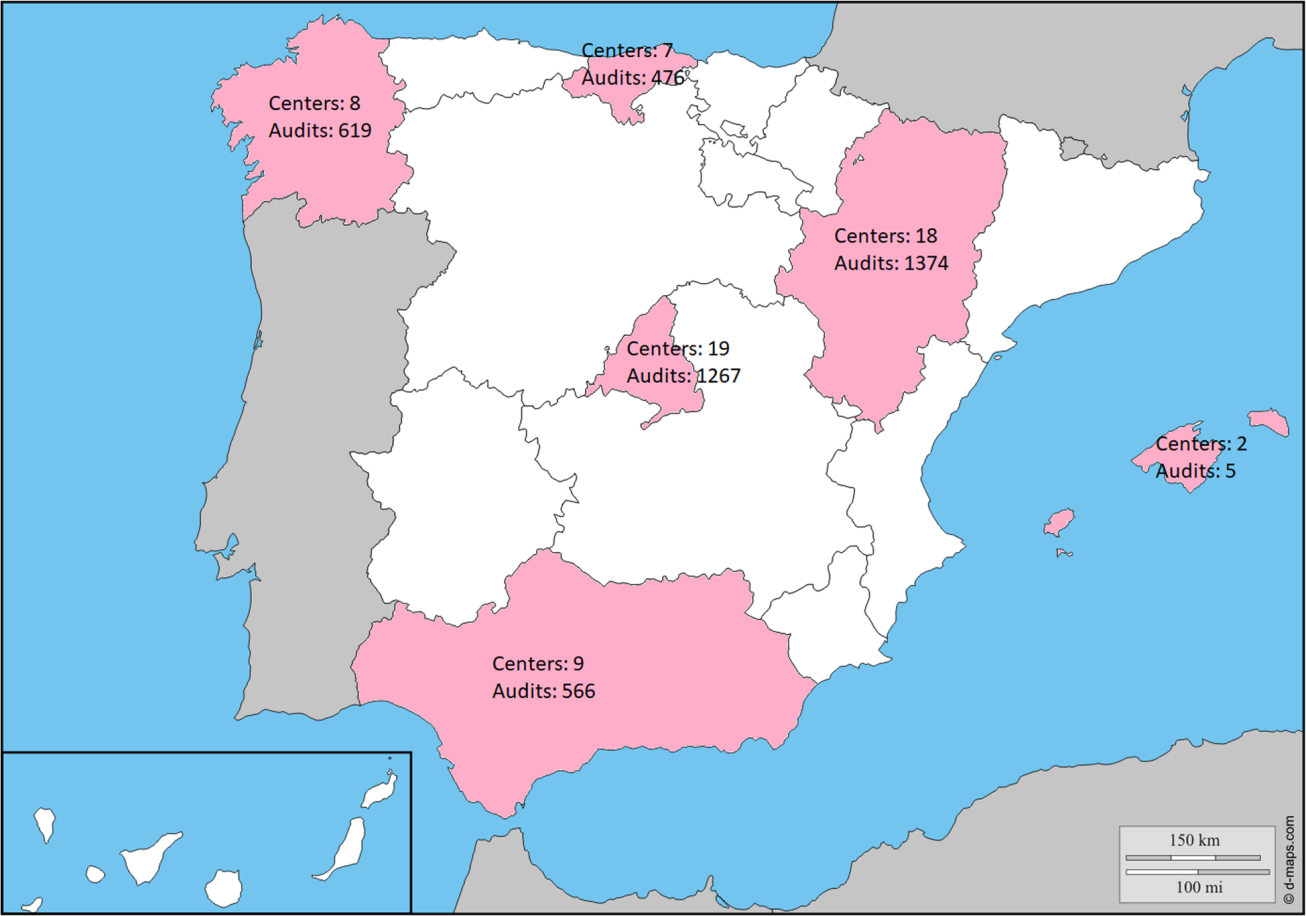
Distribution of audited cases throughout the country, Map obtained from http://d-maps.com/carte.php?num_car=2208&lang=es

During the study, 4307 cases from 63 PCC in 6 regions of the country were finally audited (Fig. 1). The description is summarized in Table 1. This was a cohort of COPD patients, with male gender predominance, in the 7th decade of life, with a considerable number of patients being active smokers and having moderate lung function impairment. Overall, there was a considerable variability among the different regions of the country and PCCs, which was significant for most variables. Interestingly, the variability of forced expiratory volume in 1 s (FEV_1_) was not significant.

**Table 1:** Characteristics of the audited cases (*n* = 4307)

Variables	Average (patient level)	Inter-center range		Inter-regional range	
		Range	*P* value ^a^	Range	*P* value ^a^
Male gender (n)	3159 (73.3)	35.7–93.8	< 0.001	67.9–78.1	< 0.001
Age (years)	71.1 (12.7)	61.9–80.2	< 0.001	67.2–73.3	< 0.001
Rural communities	1771 (41.1)	–	–	5.8–74.6	< 0.001
Active smoker (n)	1152 (26.7)	4.8–80.8	< 0.001	20.7–41.9	< 0.001
Ex-smokers (n)	1127 (26.2)	6.1–40.0	< 0.001	0.0–76.5	< 0.001
Life-long never smokers (n)	579 (13.4)	0.0–84.6	< 0.001	8.3–19.0	< 0.001
Previous smoking status unknown (n)	1449 (33.6)	16.8–51.6	< 0.001	2.2–88.1	< 0.001
Tobacco history (pack-years)	44.2 (48.6)	9.0–100	< 0.001	29.9–48.8	0.045
Comorbidities (Charlson index)	2.2 (1.4)	1.5–3.2	< 0.001	1.9–2.4	< 0.001
Comorbidities (COTE index)	1.1 (1.8)	0.5–1.8	< 0.001	0.8–1.4	0.003
Comorbidities: cardiac ^b^ (n)	1280 (29.7)	13.3–63.6	< 0.001	26.5–32.5	0.076
Comorbidities: vascular ^c^ (n)	913 (21.2)	0–40.0	< 0.001	15.7–24.9	< 0.001
Comorbidities: neoplasms (n)	476 (11.1)	0–31.3	< 0.001	2.9–15.4	< 0.001
Comorbidities: sleep apnea (n)	201 (4.7)	0–20.3	< 0.001	0–7.8	< 0.001
Comorbidities: eyedrops use (n)	54 (1.3)	0–18.2	< 0.001	0.9–2.8	0.018
Comorbidities: psychiatric drugs ^e^ (n)	501 (11.6)	0–38.2	< 0.001	6.8–15.8	< 0.001
Comorbidities: prostatic hyperplasia (n)	725 (16.8)	0–33.3	< 0.001	8.8–21.7	< 0.001
Body mass index (kg/m^2^)	29.2 (5.5)	26.7–37.1	< 0.001	28.4–30.9	0.004
Current FEV_1_ (mL) ^d^	1690 (694)	810–2720	0.115	1640 – 1794	0.744
Current FEV_1_ (%) ^d^	64.3 (22.8)	39.0–97.0	0.307	21.0–25.7	0.136
Current FEV_1_/FVC (%) ^d^	62.3 (13.2)	69.0–51.0	0.741	60.2–65.1	0.249

Data are expressed as mean (standard deviation) or absolute (relative) frequencies, as needed

*FEV*_*1*_ forced expiratory volume in the first second

^a^ Calculated by Chi-squared test or ANOVA to test for variability

^b^ Includes coronary artery disease, heart failure, and atrial fibrillation or flutter

^c^ Includes coronary artery disease, peripheral vascular disease, and cerebrovascular disease

^d^ Obtained from post-bronchodilator spirometry, and if not available, from pre-bronchodilator spirometry

^e^ Includes anxiolytics, antidepressants, and antipsychotics

The results of the clinical audit in terms of diagnostic and clinical evaluation are summarized in Table 2. The diagnoses were correct in a minority of cases. In the audited visit, only 67 (1.6%) cases had a current post-bronchodilator obstructive spirometry. If the diagnostic spirometry was included, 184 (4.3%) cases had a post-bronchodilator obstructive spirometry either in the audited visit or in the initial diagnostic visit. However, when the inter-regional and inter-center ranges were analyzed, this result improved in some centers, although none of them reached the threshold of 80% considered to be excellent. The consideration of symptoms as an added criterion for assessing the diagnoses worsened the results since a considerable number of audited cases did not record chronic symptoms. With considerable variability, dyspnea, chronic bronchitis, and asthma symptoms were not adequately registered in most audited cases, although some centers performed than others. Interestingly, exacerbations were more frequently registered in as many as 80% of the cases audited.

**Table 2:** Audit results from the diagnostic and evaluation perspective (*n* = 4307)

Variables	Average (Patient level)	Inter-center range		Inter-regional range	
		Range	*P* value ^a^	Range	*P* value ^a^
Final diagnosis correct					
Exposure + obstruction ^b^	758 (17.6)	0–60.0	< 0.001	9.8–23.3	< 0.001
Exposure + obstruction ^b^ + symptoms	369 (8.6)	0–41.3	< 0.001	0.7–14.0	< 0.001
Evaluation of the clinical presentation					
Dyspnea registered	820 (19.0)	0–94.9	< 0.001	2.7–31.8	< 0.001
Dyspnea registered by mMRC	479 (11.1)	0–77.8	< 0.001	2.5–21.4	< 0.001
Cough and sputum registered	1099 (25.5)	0–94.4	< 0.001	0.9–49.6	< 0.001
Sputum color registered (*n* = 789 with chronic bronchitis)	358 (45.4)	0–77.9	< 0.001	19.2–77.0	< 0.001
Asthma symptoms registered	856 (19.9)	0–86.7	< 0.001	0.2–35.3	< 0.001
Exacerbations in the previous year registered	3507 (81.4)	2.6–100	< 0.001	62.0–95.6	< 0.001
Evaluation of therapies					
Current smoking status registered	1874 (43.5)	0–100	< 0.001	18.2–66.1	< 0.001
Exercise registered	1950 (45.3)	0–100	< 0.001	13.9–74.5	< 0.001
Influenza vaccination registered	3777 (87.7)	43.9–100	< 0.001	79.1–96.6	< 0.001
Pneumococcal vaccination registered	2991 (69.4)	2.5–100	< 0.001	31.5–95.0	< 0.001
Adverse effects registered	173 (4.0)	0–100	< 0.001	1.6–6.9	< 0.001
Treatment adherence registered	610 (14.2)	0–94.9	< 0.001	5.3–27.4	< 0.001
Inhaler satisfaction registered	195 (4.5)	0–47.6	< 0.001	0.8–6.9	< 0.001
Evaluation of complementary tests used					
Spirometry solicited	520 (12.1)	0–80.0	< 0.001	4.7–23.7	< 0.001
COPD assessment test administered	46 (1.1)	0–18.8	< 0.001	0–2.6	< 0.011
Sputum culture solicited	43 (1.0)	0–21.7	< 0.001	0–3.9	< 0.001
Alpha1-antitrypsin solicited (*n* = 4199 with no previous determination)	5 (0.1)	0–7.7	< 0.001	0–0.2	0.896
Alpha1-antitrypsin solicited at any time in the clinical record	114 (2.6)	0–23.8	< 0.001	0.4–7.4	< 0.001

Data are expressed as absolute (relative) frequencies in comparison to the whole cohort unless otherwise specified

*mMRC* modified Medical Research Council

^a^ Calculated by Chi-squared test or ANOVA to test for variability

^b^ Spirometric obstruction detected by post-bronchodilator spirometry and, if not available, by the most recent pre-bronchodilator spirometry

The most frequently registered non-pharmacological therapies were vaccinations. Interestingly, current smoking habit was registered in 43.5% of audits, similar to the value for registered exercise. Three other key aspects of non-pharmacological evaluation, namely treatment adherence, adverse effects, and inhaler satisfaction, were rarely registered.

Regarding the complementary tests, spirometry was performed in a minority of cases. Alpha 1-antitrypsin determination was the only item showing no significant variability among regions, and this was anecdotal. Other tests and assessments including computed tomography scan and sputum eosinophils, as well as health-related quality of life, were more rarely obtained from the medical records (data not shown).

**Fig. 2:**
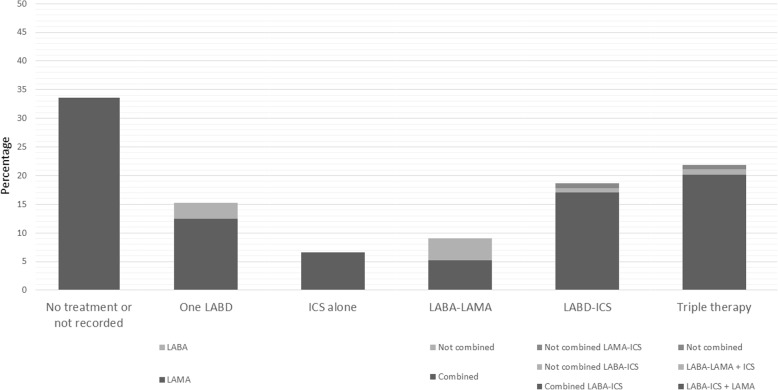
Distribution of maintenance inhaled therapies. ICS: inhaled corticosteroids; LABA: long-acting ß_2_ agonist; LABD: long-acting bronchodilator; LAMA: long-acting muscarinic antagonist

**Fig. 3:**
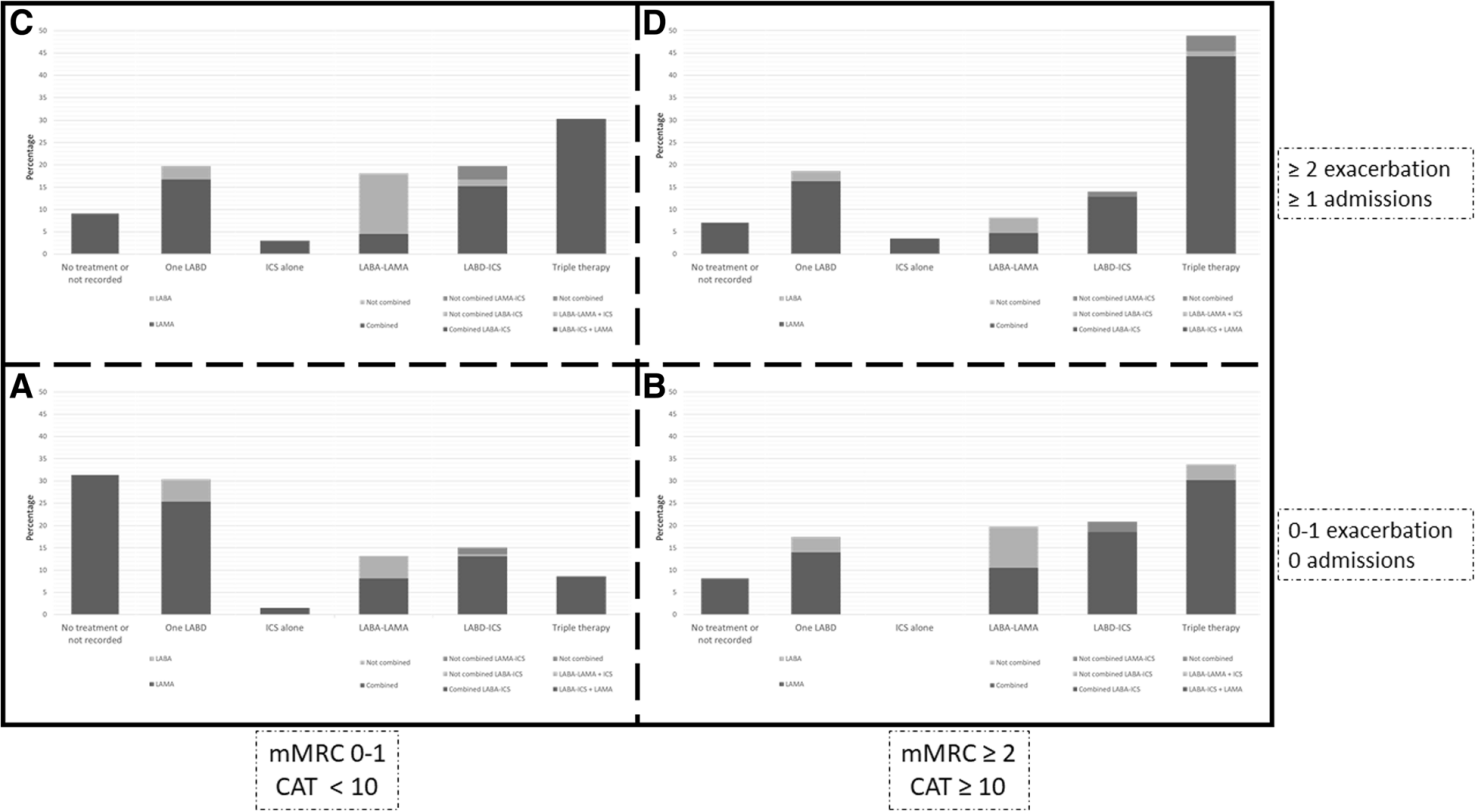
Distribution of maintenance inhaled therapies according to GOLD 2017 groups. Only in 436 cases out of the total 4307 cases audited (10.1%), GOLD classification could be assessed, based on exacerbation frequency and symptoms. ICS: inhaled corticosteroids; LABA: long-acting ß_2_ agonist; LABD: long-acting bronchodilator; LAMA: long-acting muscarinic antagonist; CAT: COPD Assessment Test; mMRC: modified Medical Research Council

The use of current therapies for COPD is summarized in Table 3. The most frequently prescribed non-pharmacological treatment was influenza vaccination. Only in 24.4% of audits was it registered that the patients had quitted smoking. The distribution of pharmacological treatment is shown in Fig. 2. Triple therapy, followed by the combined use of an ICS and a LABD, was most commonly used during the audit period. In the whole cohort, 40 (0.9%) cases were receiving non-combined ICS-LAMA therapy. GOLD 2017 patient types were identified in 436 (10.1%) cases, and the distribution of inhaled therapies in this group of patients is shown in Fig. 3. Altogether, the treatments including an ICS by GOLD 2017 patient types were: GOLD A 50 (25.3%), GOLD B 47 (54.7%), GOLD C 35 (53.0%), and GOLD D 57 (66.3%). The percentage of GOLD 2017 patient types who did not receive any maintenance inhaled therapies was: GOLD A 31.3%, GOLD B 8.1%, GOLD C 9,1%, GOLD D 7.0%.

**Table 3:** Audit results regarding administration of treatments (*n* = 4307)

Variables	Average (Patient level)	Inter-center range		Inter-regional range	
		Range	*P* value ^a^	Range	*P* value ^a^
Non-pharmacological treatments					
Recommendations about not smoking	1051 (24.4)	0–76.5	< 0.001	6.1–36.4	< 0.001
Perform some exercise	958 (22.2)	0–84.7	< 0.001	3.2–41.8	< 0.001
Influenza vaccination administered	2869 (66.6)	34.1–95.2	< 0.001	53.4–73.3	< 0.001
Pneumococcal vaccination administered	1620 (37.6)	0–82.3	< 0.001	10.8–65.2	< 0.001
Inhaled maintenance therapies					
No inhaled treatment/not available	1446 (33.6)	8.8–54.9	< 0.001	26.5–42.9	< 0.001
One long-acting bronchodilator	653 (15.2)	0–33.3	< 0.001	11.3–18.9	< 0.001
LAMA + LABA ^b^	392 (9.1)	0–35.3	< 0.001	6.9–10.0	0.025
ICS alone	67 (1.6)	0–11.1	0.018	0.6–3.4	0.002
ICS + one long-acting bronchodilator	804 (18.7)	0–38.2	< 0.001	14.8–25.4	< 0.001
Triple therapy	945 (21.9)	0–50.0	< 0.001	18.9–24.9	0.002
ICS-containing regimens	1816 (42.2)	12.5–66.3	< 0.001	38.6–51.1	< 0.001
Incorrect prescription ^c^	224 (5.2)	0–14.7	< 0.001	3.6–6.9	< 0.001
Oral maintenance therapies					
Roflumilast	58 (1.3)	0–7.7	< 0.001	1.0–1.7	0.693
Mucolytics	96 (2.2)	0–26.7	< 0.001	0.2–4.0	< 0.001
Antibiotics	29 (0.7)	0–6,7	< 0.001	0.2–1.3	0.022
Methylxanthines	97 (2.3)	0–17.6	< 0.001	0.2–6.2	< 0.001
Home-based therapies					
Long-term oxygen therapy	175 (4.1)	0–18.8	< 0.001	2.3–5.4	0.029
Home mechanical ventilation	35 (0.8)	0–10.0	< 0.001	0.1–1.9	< 0.001
Nebulized therapy	121 (2.8)	0–22.4	< 0.001	0.1–6.4	< 0.001

Data are expressed as absolute (relative) frequencies in reference to the whole cohort unless otherwise specified

*LABA* long-active ß_2_ agonist, *LAMA* long-acting muscarinic antagonist, *ICS* inhaled corticosteroids

^a^ Calculated by Chi-squared test or ANOVA to test for variability

^b^ Combined in one single inhaler or not. Only indacaterol-glycopyrronium combination was available in the country at the time of the audit

^c^ Per the protocol, the following scenarios were deemed to represent incorrect prescriptions: GOLD 2017 B-D patients without any medication registered, any case of ICS use alone, and those prescriptions that duplicated drugs in combined or single therapies

## Discussion

The results of this study indicate that there is considerable variability in clinical performance that cannot completely be attributed to the severity of the disease. Most evaluated parameters were judged to fall under inadequate performance, except two (i.e., registration of influenza vaccination, and registration of exacerbations in the previous years) that were classified as excellent.

There is increasing evidence and awareness that patients with various health problems do not consistently receive the recommended care despite the multitude of clinical practice guidelines and there is a gap between the clinical care provided and that recommended by practice guidelines [29]. In this regard, clinical audits constitute a valuable tool to assess clinical performance and set the basis for future improvement. The present study represents an innovative initiative to perform a national clinical audit of COPD care in primary care in Spain, as in other countries (https://www.rcplondon.ac.uk/COPD).

The diagnosis of COPD is of utmost importance. The latest version of the GOLD criteria requires three main components for a correct diagnosis including respiratory symptoms, previous exposure to noxious gases and particles, and obstruction on post-bronchodilator spirometry. Our results showed that in the primary care setting, some of these three criteria were missing in a high proportion of patients. Even if older versions of the GOLD criteria are applied (which do not categorically state the requirement of symptoms as a diagnostic sub-criterion) and the results of pre-bronchodilator spirometry in the absence of post-bronchodilator spirometry results are used, the number of acceptably diagnosed cases would still be low, only on the basis of previous exposure and the presence of obstruction. In this context, the availability and use of spirometry in primary care is essential. In the UK, a recent analysis pointed out that although the quality of spirometry procedure undertaken in primary care is high, this does not reflect the quality of interpretation, suggesting an unmet training target in primary care [19]. In Spain, although most centers received training for conducting spirometry tests, this was routinely done in less than 40% of centers and the mean number of spirometries conducted per week in the primary care settings was 5.6 (ranging from 2.0 to 8.8 depending on the region) [30]. In the US, spirometry is infrequently assessed even in those more severe patients receiving triple therapy [31]. Interestingly, the performance is better in other countries [18]. The consequences of this incorrect diagnosis impact several domains, with many patients receiving pharmacological treatment that is not needed and a potential for drug-related adverse effects, giving health services to wrong patients, subjecting patients to additional and often unnecessary tests, labelling them as sick or at-risk, telling them to modify their daily living habits, or insisting on monitoring them regularly. Additionally, it also impacts the health system, leading to potential extra costs [32]. Consequently, in the light of our results, the clinician should be aware of the diagnostic criteria for COPD and consider respiratory symptoms, exposure to risk factors and spirometry results before firmly establishing this diagnosis.

In 33.6% of cases, there was no information on maintenance inhaled therapies. This finding has been previously reported but with a considerable difference. A recent analysis of the medical records of 3376 patients from a general practice in Denmark revealed that 74.4% of them did not receive any maintenance inhaled medication even after the spirometric diagnosis of COPD [33]. In the United Kingdom 28% of the 20,154 patients whose medical records were analyzed [20], received no initial pharmacological treatment. The number was reported to be as low as 20% in another study, which looked into the records of of 29,815 patients [34]. In Sweden, this figure was far below 10% in secondary care [35]. In the United States, it has been reported that 55% of such patients did not receive inhaled maintenance therapy [36]. The reasons for this finding fall outside the scope of the present study and would require a specific debate and an ad-hoc investigation.

Double bronchodilation was probably underestimated during the period of the audit, as double bronchodilator therapy was not widely available at the time. Only one (indacaterol-glycopyrronium) of the 4 double bronchodilator medications later approved in Europe for the treatment of COPD [37] was available in Spain at that time. In spite of this, the number of patients receiving LABA-LAMA combination therapies was slightly higher than those on single therapies, suggesting that the use of LABA-LAMA combinations may have continued to increase in subsequent years. A fact which was confirmed in subsequent audits [16].

Previous studies have reported the overuse of ICS, as well as an increase in their use for all GOLD patient types. Price et al. have reported the use of ICS in 50% of COPD patients in primary care settings in the United Kingdom [38]. In the present study, ICS-containing regimens were used in approximately 42.2% of all patients, ranging from 25.3% in GOLD 2017 A to 66.3% in GOLD 2017 D. Most patients receiving triple therapy were on a LABA-ICS + LAMA combination. Although the efficacies of both forms of triple therapy (LABA-ICS + LAMA vs. LABA-LAMA + ICS) may be similar [39], their appropriateness for use may have changed now in the light of recent studies and recommendations [40, 41]. Interestingly, a recent real-world prescription analysis in the UK, demonstrated inappropriate prescribing of triple therapy and confirmed that starting patients on ICS plus LABA results in the inevitable drift to overuse of triple therapy [42]. Although in the present study only 10% of cases could be classified according to GOLD 2017 patient types, this scenario represents an improvement from previous reports on the use of ICS in COPD. Recent studies have shown that ICS prescription in GOLD A/B patients is common, with significant regional variation independent of lung function impairment [34]. Despite the reported overuse of triple therapy [42, 43], we found an increase in their prescription with the progression of GOLD 2017 patient types. Notably, although not explicitly recommended in the guidelines, some patients were receiving LAMA-ICS combination therapy. Only a few trials have evaluated this combination in COPD patients [44, 45].

Overall, there was a considerable variability among the different regions of the country and PCCs, which was significant for the majority of variables due to the large sample size. Interestingly, the variability of FEV_1_ was not significant, suggesting that the variability found is not related to disease severity as measured by this parameter. Previous studies have demonstrated significant variability in the processes and outcomes of COPD care in different settings [46, 47]. It has been shown that this variability in clinical practice is not exclusively influenced by clinical presentation or the resources available, but that there is a so-called cluster effect [15]. The cluster effect indicates that patients with similar characteristics may experience different processes of care and outcomes, depending on the center because they are subject to distinct common contextual influences, beyond resources or standards of care, like the characteristics of the catchment area, such as socioeconomic status, and utilization of health services or COPD specific criteria in a determined clinical situation. In our audit we found an extremely important variability in clinical practice that should be explored in the future. The idea behind quality of care is that all patients with the same condition should be managed similarly. However, this is very complex in clinical practice since the clinical presentation of chronic diseases is variable, the perception by the patients varies and is influenced by many personal aspects, and the response to therapies is also variable. Therefore, describing the variability is essential to really understand its potential implications allowing us to seek specific approaches in the future.

Clinical audits have gained traction in healthcare systems as a way of obtaining information on the clinical care being provided, as shown by a recent qualitative study in a primary care setting [18]. This information is of interest to both funding bodies, who want to ensure that the care they finance is of the highest possible standard, and for patients who hope to receive safe and effective healthcare. However, despite evidence from several studies on audit and feedback, few data are currently available on how to use this information. A recent systematic review assessed the effectiveness of audit and feedback and reported an inconsistent picture; some evaluations obtained positive results, whereas others did not [48]. In this context, feedback from the audited information constitutes a key step in improving clinical practice [49]. Probably the most extended initiative is that of care bundles that have been successfully applied in health care [50]. The applicability of these care bundles or any other initiative aiming to improve primary health care in COPD patients should be assessed next.

The strengths of this project are the potential to raise the profile of COPD, and provide an opportunity to promote respiratory medicine in the community, offer suggestions and recommendations on organization of care for the future COPD management guideline, and develop educational resources to support improved clinical practice in all areas, especially those identified as poor practice. Also, the audit provides a formal documentation of the difference between recommended best practices and the status quo, thereby identifying areas requiring improvement.

To interpret the results correctly, several key aspects of the methodology must be considered. First, the audit assessed interventions that had already been performed, and not the results thereof. For instance, it assessed if the physician in charge registered the degree of dyspnea, rather than the intensity of dyspnea after intervention. Thus, the aim of the audit was not to have an idea of the clinical situation of the patient but to have an idea of the clinical performance of the practitioner. Second, the clinical audit made its evaluation from the contents of medical records. Therefore, what was not documented was not evaluated. This does not imply that no intervention had taken place. Accordingly, the estimates presented here were those inferred from the medical record and we may have underestimated the actual figures. The need to record every aspect of clinical practice in the medical record has been previously emphasized [51], and our results further ascertain such need Third, although medical records are electronic in all participating PCC, the application and interface to access the data were different in different regions of the country. Thus, different investigators interacted with different systems, which may have influenced the availability of the data. To check for the quality of these data, the values that were extreme, missing, or inconsistent were returned to the investigators for correction. Fourth, the concept of incorrect prescription could be questionable or misleading is based on the GOLD document. GOLD patient types constitute a way of categorizing patients to ease the selection of the therapeutic approach [40]. However, the magnitude of the impact of the disease can vary considerably within one type [52]. Also, patients with specific clinical phenotypes or presentations might be classified under the same GOLD type, and therefore warrant a change in therapy [53]. Therefore, to confirm those cases with incorrect prescriptions, we selected those cases with specific criteria showing incorrect treatment strategies. Finally, the present paper describes great variability in clinical practice. The next issue to solve would be to do an analysis of the confounding or explanatory variables for such a wide range of variables, which will be a matter of another analysis.

In summary, our results show areas of greatest concerns in COPD care. Clinicians should be aware that only a correct diagnosis makes it possible to select proper pharmacological treatment for COPD, that COPD cannot be diagnosed without proving irreversible obstructive pattern on post-bronchodilator spirometry, that cigarette smoke exposure and especially duration of smoking in years is an important risk factor for the disease and should be systematically evaluated, that smoking cessation, physical activity, nutrition, vaccinations as well as recognition and treatment of comorbidities are key in the management of the disease beyond the use of bronchodilators, and that the use of the different pharmacological approaches should be patient-tailored, and confined to those who do get the benefit from them with no harm.

## Conclusions

In conclusion, the present analysis, to the best of our knowledge, is the first ever clinical audit carried out in SpanishPCC to evaluate clinical performance in stable COPD patients. The results show that there is a considerable variability in clinical performance not fully attributable to the severity of the disease and that the majority of the evaluated parameters fell in the range of inadequate performance. A study of the determinants of this variability will help us to understand clinical behavior, and establish strategies to strengthen the clinical practice of COPD management in primary care.

## Abbreviations

COACH: Community Assessment of COPD Health Care
COPD: chronic obstructive pulmonary disease
FEV_1_: forced expiratory volume in 1 s
GesEPOC: Spanish National Guideline for COPD
GINA: Global Initiative for Asthma
GOLD: Global Initiative for Obstructive Lung Disease
GRAP: Spanish Primary Care Respiratory Group
ICS: inhaled corticosteroids
LABA: long-acting ß_2_ agonist
LABD: long-acting bronchodilator
LAMA: long-acting muscarinic antagonist
PCC: primary care centers
SEPAR: Spanish Society of Pneumology and Thoracic Surgery
